# Lean nonalcoholic fatty liver disease and sarcopenia

**DOI:** 10.3389/fendo.2023.1217249

**Published:** 2023-06-23

**Authors:** Milian Chen, Ying Cao, Guang Ji, Li Zhang

**Affiliations:** Institute of Digestive Diseases, Longhua Hospital, Shanghai University of Traditional Chinese Medicine, Shanghai, China

**Keywords:** lean non-alcoholic fatty liver disease, sarcopenia, insulin resistance, visceral obesity, metabolic inflammation

## Abstract

Nonalcoholic fatty liver disease (NAFLD) has become one of the most common chronic liver diseases in the world. The risk factor for NAFLD is often considered to be obesity, but it can also occur in people with lean type, which is defined as lean NAFLD. Lean NAFLD is commonly associated with sarcopenia, a progressive loss of muscle quantity and quality. The pathological features of lean NAFLD such as visceral obesity, insulin resistance, and metabolic inflammation are inducers of sarcopenia, whereas loss of muscle mass and function further exacerbates ectopic fat accumulation and lean NAFLD. Therefore, we discussed the association of sarcopenia and lean NAFLD, summarized the underlying pathological mechanisms, and proposed potential strategies to reduce the risks of lean NAFLD and sarcopenia in this review.

## 1 Introduction

Nonalcoholic fatty liver disease (NAFLD), also known as metabolic-associated fatty liver disease (MAFLD), is the most common chronic liver disease in the world (1), which composed of a series of progressive liver diseases, including NAFL, non-alcoholic steatohepatitis (NASH), and fibrosis (2). Lean NAFLD has been an emerging topic of the current clinical study, given a considerable portion of NAFLD patients without obesity and its distinguishing courses of disease and manifestations (3–5). Lean NAFLD is usually defined by Body Mass Index (BMI), with BMI <23kg/m^2^ in Asians and <25kg/m^2^ in European and American (6). An epidemiological survey showed that the global prevalence of NAFLD has risen to 32.4% (7). Although obesity is a major contributing factor to NAFLD, approximately 40% of patients with NAFLD are nonobese and 20% are lean (8). Lean MAFLD is often occurred in old people, with a higher proportion of females (9). The study by Younossi et al. (10) found that the prevalence of lean NAFLD varies among different ethnic groups, ranging from 5%- 45% in Asians to 5%- 20% in Europeans. Among 6905 Chinese people with BMI<25kg/m^2^, 7. 27% had hepatic steatosis; In another study, 18% of 2000 Chinese residents with BMI<24kg/m^2^ were diagnosed with NAFLD (11). This may be related to regional differences in dietary habits (including high fructose, low protein and dietary fiber intake (11–14) and lifestyle (such as sedentary, lack of physical exercise and sleep habits) (15–17).

## 2 Clinical features and risks of lean NAFLD

Obesity is one of the major characteristics of NAFLD, and is known as a risk factor for the development of NAFLD (6). People with normal BMI are often overlooked in screening for NAFLD. Moreover, lean NAFLD is often considered to be benign in contrast to obese NAFLD, even images show identical steatosis (18). Chen et al. (3) included 538 patients with NAFLD confirmed by liver biopsy, and found that compared with non-obese patients, lean patients had better metabolic status and lower incidence and severity of T2DM and metabolic syndrome. But these differences occur in the early stage of liver disease, and the advanced liver disease progression of lean NAFLD isn’t improved (3). This suggests the homeostasis in lean NAFLD is gradually destroyed and subsequent progression can’t be avoided, despite the baseline metabolic and histological status of them being better than non-lean subjects (3).

HOMA-insulin resistance (Homeostasis model assessment-insulin resistance) is significantly higher among the lean NAFLD group in comparison to the obese group in a study by Parth et al. (19). And multiple studies have found that both non-obese and lean NAFLD have long-term hepatic and extrahepatic complications (10, 11). This may explain why some studies have reported higher advanced liver disease severity and mortality in lean NAFLD than in non-lean NAFLD. Lean NAFLD patients have a higher risk of metabolic syndrome and all-cause mortality in comparison to obese NAFLD patients (12, 18–21). This suggests that lean NAFLD is not a metabolically healthy status. Lean NAFLD patients have the same pathological features as obese NAFLD, and the same or even shorter course of progression to NASH (22). Numerous studies have reported that lean NAFLD patients showed metabolic risk factors, such as dyslipidemia, arterial hypertension, insulin resistance and type 2 diabetes mellitus (T2DM), which are closely related to the severity of liver disease (12, 23, 24). A large, prospective, community-based cohort (25) provides evidence that lean NAFLD subjects have more progressive liver disease and poorer clinical outcomes, independent from the usual risk factors and lifestyle. This is confirmed in another meta-analysis on mortality and liver events in patients with lean NAFLD (20), as well as in a cohort study in China (21).

Central obesity is a typical characteristic of lean NAFLD (26). Unlike subcutaneous fat storage, excessive visceral fat leads to the accumulation of pro-inflammatory cytokines including tumor necrosis factor-α (TNF-α) and interleukin-6 (IL-6), and reduction of many anti-inflammatory factors such as adiponectin, which promotes metabolic inflammation (27, 28). Visceral fat is reported to be more closely associated with metabolic syndrome than subcutaneous fat (28, 29). The proportion of central obesity of lean NAFLD is observably increased, despite the BMI of lean NAFLD patients is in the normal range or even lower (23). It is reported that the waist circumference and total abdominal fat levels in the non-obese group were lower than those in the obese group, but there was no prominent difference in visceral fat levels between the two groups. In some non-obese patients, the visceral fat area even exceeds 100cm^2^, which is higher in contrast to that in obese patients (30). Therefore, excessive visceral fat might be a driving factor for the disease progression of lean NAFLD.

## 3 Association between lean NAFLD and sarcopenia

Skeletal muscle as a locomotor organ as well as a metabolic organ accounts for 40-50% of lean body mass in adults, which plays one of the most crucial roles in the control of energy metabolism (31). Sarcopenia, previously considered an aging-related syndrome involving a decrease in muscle quantity and quality as well as physical performance, is now recognized as a progressive pathological process associated with type 2 diabetes mellitus (T2DM), metabolic syndrome, liver disease, and cardiovascular disease (32). It is primarily associated with aging and secondarily with diseases mediated by systemic inflammation and insulin resistance (32).

There is a close relationship between skeletal muscle and the liver, in particular, in terms of glucose, amino acid, and ammonia metabolism (33). In a Korean epidemiological study of 3305 patients with metabolic syndrome, 739 (22. 4%) had sarcopenia (34). And the coexistence of NAFLD and sarcopenia have been reported in a series of clinical studies (**Table 1**) (35–44). NAFLD patients with sarcopenia had a 2-fold higher risk of developing NASH and significant fibrosis independent of obesity and insulin resistance (45). Lean NAFLD patients are especially prone to sarcopenia (46). The prevalence of sarcopenia is significantly increased in patients with NASH compared to that in healthy subjects (35.0% versus 8.7%) (45). A recent study demonstrated that NAFLD developed in 14.8% of its participants during a 7-year follow-up, with an increased incidence in participants with the lowest tertile of skeletal muscle mass at baseline (47). Sarcopenia is associated with poor clinical outcomes, including severe hepatic fibrosis and increased mortality, in NAFLD patients (48–51). Therefore, low skeletal muscle mass may be an important risk factor for NAFLD, especially lean NAFLD. In addition, sarcopenia is also associated with cirrhosis and is a prognostic determinant of cirrhosis (33, 52).

**Table 1:** The association between sarcopenia and NAFLD.

Study	Study attribute	Study objects	Assessment of sarcopenia	Assessment of NAFLD	Findings	Ref.
Lee et al. (2015)	Cross-sectional cohort.	15,132 subjects of Korean	DXA	Noninvasive models	The risk of NAFLD in patients with sarcopenia increased 2.3- to 3.3-fold	(35)
Kim et al. (2021)	Longitudinal cohort.	11,065 subjects of American	BIA	US	Sarcopenia was associated with a higher risk for all-cause mortality. patients with both sarcopenia and NAFLD had a higher risk for all-cause mortality.	(36)
Chung et al. (2019)	Cross-sectional cohort.	5,989 subjects of Korean	BIA	US	Sarcopenia was significantly associated with the presence and the severity of NAFLD	(37)
Golabi et al. (2020)	Cross-sectional cohort.	4,61 1 subjects of American	Appendicular lean mass divided by body mass index	US	Sarcopenia is associated with increased mortality amongst NAFLD patients, which should be a part of clinical assessment of patients with NAFLD.	(38)
Petta et al. (2017)	Cross-sectional cohort.	225 subjects of Italian	BIA	Liver biopsy	The risk of fibrosis in NAFLD in patients with sarcopenia increased 2-fold	(39)
Zhai et al. (2018)	Cross-sectional cohort.	494 subjects of Chinese	DXA	US	NAFLD is significantly associated with sarcopenia.	(40)
Wijarnpreecha et al. (2019)	Cross-sectional cohort.	11,325 subjects of American	BIA	US	The risk of advanced fibrosis in patients with sarcopenia increased 1.8-fold.	(41)
Hsieh et al. (2021)	Cross-sectional cohort.	521 subjects of Korean	CT	Liver biopsy	The risk of fibrosis in NAFLD with sarcopenia increased	(42)
Hsieh et al. (2022)	Longitudinal cohort.	338 subjects of Korean	CT	Liver biopsy	Muscle loss is significantly associated with progression of NASH in patients with sarcopenia	(43)
Harring et al. (2023)	Cross-sectional cohort.	5,856 NHANES participants	FNIH definition	Transient elastography	Patients with sarcopenic NAFLD are at risk for significant fibrosis and advanced fibrosis.	(44)

DXA, dual energy X-ray absorptiometry; CT, computed tomography; BIA, Bioelectric impedance analysis; US, ultrasonography; FNIH, Foundation for the National Institutes of Health; NAFLD, nonalcoholic fatty liver disease; NASH, nonalcoholic steatohepatitis; NHANES, national health and nutrition examination survey.

## 4 Potential mechanisms of lean NAFLD and sarcopenia

### 4.1 Dietary intake and lean NAFLD

The pathophysiology of lean NAFLD can be impressed by diet and nutrients (53, 54). A matched case-control study included 351 Chinese adults, and found the average weekly exercise and daily sleep duration of lean NAFLD people were lower than that of healthy lean NAFLD people, while the average daily intake of total calories, carbohydrate, total cholesterol, fat and protein was higher than that of healthy lean NAFLD people (55). Total starchy foods intake was higher in those with NAFLD compared to the lean healthy group, but no significant difference was found among lean and non-lean NAFLD participants regarding nutrients and food items, indicating that the chance of lean NAFLD would increase by an increase in carbohydrate, potato, and fat intake (56). But in the study of Younossi et al. (57), no difference was found in macronutrients intake (carbohydrate, fat, and protein), vitamins, and minerals between lean NAFLD subjects and lean healthy controls. They suggested that this may indicate that NAFLD in lean patients has occurred due to other metabolic abnormalities. Others have postulated that the cause of lean NAFLD could be due to genetic characteristics, impaired intestinal motility, and some other metabolic disturbances that are not related to weight status (58). Since studies on the association between lean NAFLD and diet are less at present, and is still controversial, it is urgent to assess dietary intakes and pattern, and diet quality with adequate sample size in lean NAFLD people to find if there is any association between them.

### 4.2 The Role of skeletal muscle and glucose metabolism

The concerted regulation of glucose uptake, utilization, and storage by tissues is critical to maintaining blood glucose homeostasis (59). Insulin increases glucose uptake into peripheral tissues, primarily skeletal muscle and adipose tissues, which express the GLUT4 isoform of the glucose transporter. In unstimulated fat or muscle cells (basal state), 3~10% of GLUT4 is located at the cell surface and >90% is in intracellular compartments (60). GLUT4 participates in a cycle that consists of exocytic movement of the transporter within post-biosynthetic vesicles of endosomal origin towards the plasma membrane, and of endocytic movement from the membrane back to the sorting endosomal system (61). GLUT4 exocytosis is regulated by Insulin-derived signals, including mobilization to the cellular periphery, vesicle tethering, docking and fusion, thereby increasing the cell-surface amount of GLUT4 and the rate of glucose uptake (62).

Skeletal muscle expresses high levels of GLUT4, responsible for approximately 80% of glucose clearance under physiological conditions (32, 46). In mice, muscle-specific knockout of GLUT4 leads to severe glucose intolerance (63). According to the morphology and metabolic features, muscle fibers are mainly divided into type I and type II subtypes. Muscles composed of different types of muscle fibers show great differences in blood glucose metabolism, and the density of glucose transporter 4 (GLUT4) in type I muscle fibers is higher than that in type II muscle fibers (64). Gaster et al. (65) found that GLUT4 expression was reduced in type I fibers in T2DM patients by immunohistochemistry and morphometry. Therefore, it can be assumed that the decrease in insulin-stimulated glucose uptake in the skeletal muscle of T2DM patients might be due to the decrease of GLUT4 in type I fibers. In addition, muscle fibers (especially type IIb muscle fibers) can ameliorate metabolic abnormalities by secreting proteins and muscle factors, while loss of skeletal muscle due to aging or underlying diseases can exacerbate glucose intolerance (66).

### 4.3 Sarcopenia and insulin resistance

Insulin resistance is an important pathogenesis of sarcopenia and NAFLD, regardless of lean type or non-lean type (67–69). Insulin resistance refers to the reduction of glucose uptake and utilization. To sustain stable levels of blood glucose, islet β cells secret excessive insulin to compensate, leading to hyperinsulinemia (70). Increased insulin content results in decreased affinity to insulin receptors thus, further exaggerating the insensitivity of insulin. Simultaneously, increased levels of adipokines and inflammatory cytokines (such as IL-1, IL-6 and TNF-α) activate NF-κB signaling pathway and promote insulin resistance (71). Meanwhile, the weakened insulin signaling transduction also affects the downstream mediator-glycogen synthase kinase-3β (GSK3β) and glycogen synthesis (72). Collectively, excessive insulin secretion, adipokines, and inflammatory factors directly or indirectly interfere with the insulin signaling pathway, and contribute to the development of NAFLD (73).

Insulin resistance leads to increased lipolysis and consequent release of free fatty acids (FFAs) from adipose tissue to the liver, which is the main contributor to the increase of *De novo* lipogenesis (DNL) (63, 66). The mechanism of insulin-mediated decrease of muscle anabolic metabolism may be related to the activation of p38 mitogen-activated protein kinase (MAPK) and mammalian target of rapamycin (mTOR)/p70S6 kinase (74). Insulin resistance-induced hyperinsulinemia also increases myostatin levels, which further reduces skeletal muscle mass (48). Insulin resistance leads to inhibition of β-oxidation, increased gluconeogenesis, increased expression of sterol regulatory element binding protein-1c (SREBP-1c), as well as increased production of FFAs, leading to accumulation of triglycerides in skeletal muscle and liver, and the long-term level of inflammation might be responsible for the disruption of environmental homeostasis in muscle cells, ultimately leading to systemic metabolic disorders (75, 76). Conversely, a 2-year follow-up cohort included 194 community-dwelling nondiabetic older adults found that loss of lower limb muscle mass is a significant risk factor for development of insulin resistance independent of obesity (77). Sarcopenic obesity patients had higher HOMA-IR index in contrast to subjects without sarcopenia, indicating that muscle mass is also a determinant for insulin sensitivity (67).

In addition, anabolic hormones such as insulin-like growth Factor1 (IGF-1) also contribute to the progression of sarcopenia and lean NAFLD (78), as well as non-lean NAFLD (68). Skeletal muscle mass loss inhibits the growth hormone/insulin-like growth factor-1 (GH/IGF-1) axis, and blocks skeletal muscle protein synthesis (35). The GH/IGF-1 axis is involved in protein metabolism and bone growth and remodeling in skeletal muscle (79). GH/IGF-1 affects carbohydrate and lipid metabolism and have opposite effects: IGF-1 stimulates glucose uptake and facilitates insulin signaling, while GH induces lipolysis, which induces insulin resistance and elevated levels of free fatty acids (FFAs) (80). IGF-1 level decreases when insulin resistance occurs, leading to lipid deposition in intramuscular and intermuscular adipose cells, increased infiltration of adipose between muscle bundles, and aggravated damage to muscle cells (81, 82). Patients with GH deficiency are often comorbidity with NAFLD, which contributes to NASH development and progression (83). Some studies also reported low levels of IGF-1 in NAFLD patients (84, 85). These indicate that GH/IGH-1 axis is another important association between sarcopenia and lean NAFLD.

### 4.4 Sarcopenia and cytokine disorders

Sarcopenia and lean NAFLD often present with systemic chronic inflammation, elevated levels of CRP and pro-inflammatory cytokines, and reduced level of anti-inflammatory cytokines (86). This is due to the high proportion of visceral adipose tissue in patients with lean NAFLD, and then more adipose cells secret inflammatory cytokines, and the muscle tissue is in a state of continuous chronic inflammation, leading to an increased risk of muscle atrophy (87). In patients with metabolic disorders, white adipocytes become hypertrophy and proliferated, and these white adipocytes are infiltrated by activated inflammatory macrophages and other immune cells (88). Contractile myoglobin expression is reduced in muscle tubes cocultured with white adipocytes from obese individuals (89). This may indicate that too much adipose tissue disrupts the living environment of muscle cells and promotes their apoptosis. Adipose tissue produces a large number of pro-inflammatory cytokines, such as TNF-α, monocyte chemotactic protein-1 (MCP-1), IL-6, and C-reactive protein, which promote chronic inflammation, decreased muscle protein synthesis, and insulin resistance (90). The increased production of systemic inflammatory molecules and inhibited production of certain adipokines (such as leptin and appetite suppressor hormone) in lean NAFLD patients, further adversely affect the liver, pancreas and skeletal muscle (75). Altered adipokine secretion leads to increased food intake, decreased energy expenditure, and decreased insulin sensitivity in muscles (90). Immune cells and adipocytes release inflammatory cytokines, which aggravate myocyte insulin resistance and further worsen sarcopenia (91). These processes can also occur in the pathogenesis of non-lean NAFLD/T2DM and sarcopenia (68, 92).

Inflammation and oxidative stress appear to be major factors in the etiology of muscle loss (93). Muscle cells are particularly vulnerable to oxidative damage because they are post-mitotic cells and are particularly prone to the accumulation of oxidative damage molecules (94). In addition, skeletal muscle accounts for a prominent portion of total oxygen consumption, increasing the inherent risk of elevated mitochondrial-derived ROS such as H_2_O_2_ (95). Increased reactive oxygen species (ROS) production and inefficient clearance in muscle cells trigger cellular senescence and eventually muscle loss (96). The imbalance of mitochondrial unfolded protein response and mitochondrial phagocytosis modified integrated stress response systems involved in the development of sarcopenia (97). Oxidative stress also increases the production and circulation of inflammatory cytokines, which promote protein degradation in muscle (98). TNF-α also induces lipid accumulation in the liver by activating DNL (71). It is reported in many rodent studies that inhibition of TNF-α attenuates muscle proteolysis, while an infusion of TNF-α increases Myo-fibrillation lysis (99, 100). This indicates that pro-inflammatory cytokines (CRP, IL-6, and TNF-α) may increase proteolysis and muscle atrophy.

The development of sarcopenia and lean NAFLD is accompanied by a decrease in some protective factors, such as adiponectin (27, 28). Adiponectin is a hormone secreted by adipose tissue that mediates glucose and lipid metabolism in insulin-sensitive tissues such as the liver and muscle (101). In the liver, adiponectin improves insulin resistance by activating AMP-activated protein kinase (AMPK), thus promoting glucose usage and fatty acid oxidation. In addition, adiponectin plays the role of anti-inflammatory by inhibiting TNF-α, improving liver steatosis and inflammation (102, 103). Fat accumulation and aging damage the signaling pathway of the GH/IGF-1 axis, leading to decreased levels of muscle synthesis (104, 105). In an animal model, NAFLD is reported to be associated with decreased muscle mass and strength as well as decreased IGF-1 levels, suggesting that decreased IGF-1 may contribute to the development of NAFLD-related sarcopenia (106).

In addition, Irisin is also involved in the progression of sarcopenia and NAFLD (107, 108). Irisin, an exercise-induced myokine, increases energy expenditure through peroxisome proliferator-activated receptor α-dependent downstream signaling and improves insulin sensitivity and hepatic steatosis by upregulating fibroblast growth factor-21 (108). It increases glucose uptake by enhancing GLUT4 translocation and FFA β-oxidation through activating AMPK in muscle cells (108). Irisin is reported to be inversely associated with the degree of hepatic steatosis and is a potential cause of sarcopenia and NAFLD (107, 109).

### 4.5 Genetic susceptibility

NAFLD is highly influenced by genetic and environmental factors, and its susceptibility, progression and risks of related complications vary greatly between individuals, and the genetic variations of NAFLD are confirmed in genome-wide association studies (GWAS) (110). It is reported that the proportion of the G allele in *PNPLA3* and the T allele in *TM6SF2* in lean NAFLD patients is significantly higher than that in non-lean NAFLD patients, and the mutations of these two alleles are highly related to the disorder of glucose and lipid metabolism (111). The *PNPLA3* rs738409 G allele is associated with higher liver-related mortality than the C allele in a nationwide population study in the United States (112). The C allele frequency of rs2279026 and G allele frequency of rs2279028 in the *TBC1D1* gene as well as the C allele frequency of rs780093 and rs1260326 in *GCKR* gene in lean NAFLD patients are lower than those in obese patients (113). A cohort study of 5387 Chinese residents (≥60 years old) revealed that the AA genotype frequency of *SOD2* gene rs4880 in patients with lean NAFLD is lower than that in lean healthy people, and rs4880 site in *SOD2* determined the susceptibility to lean NAFLD (114). The *PEMT* gene controls liver triglyceride secretion in the form of VLDL and is associated with NAFLD. Bale et al. (115) sequenced the intact exon region of lean NAFLD patients and found that the *PEMT* rs7946 variant is associated with a three-times increase in the risk of lean NAFLD.

Variation in skeletal muscle traits among individuals can be attributed to both genetic and environmental factors (116). Though the influence of environmental factors, such as physical activity and diet, have been broadly investigated, only a few studies have identified the specific genetic influences on skeletal muscle traits (117). Sarlo et al. enrolled 120 Italian healthy women and found a positive correlation between sarcopenic normal-weight subjects and G/A-308 *TNF-α* polymorphism (118). The gene *TP53*, encoding p53, is reported to be involved in the pathogenesis of NAFLD (119). It is also affects myoblast differentiation in severe and rapid skeletal muscle atrophy, which represents a hallmark of cachexia and sarcopenia during aging (120). The G/G genotype of *TP53* codon 72 in exon 4 polymorphism is found to increase the risk of sarcopenia up to 20% (121). The skeletal muscle mass is reported to be associated with *FTO* (Fat mass and obesity-associated) genotype variation. In a study enrolled 559 non-athlete subjects, TT genotype and T allele carriers of the *FTO* rs9939609 variant had greater total body (4.8% and 4.1%) and total appendicular lean mass (3.0% and 2.1%) compared to AA genotype (122). The *FTO* genotype variants also play an important role in lipid-related parameters (123). In a cohort study, AA genotype of the *FTO* rs9939609 is found to be associated with LDL levels lean NAFLD subjects but not in overweight and obese NAFLD subjects (113). Besides, the *ACTN3*, *ACE, VDR*, *IGF1/IGFBP3*, *APOE*, *CNTF/R* and *UCP2/3* gene polymorphism are also found to be associated with muscle phenotypes (124–130), but their association with lean NAFLD is still to be determined. Therefore, certain genetic factors might be associated with both sarcopenia and lean NAFLD, but further studies are needed to verify the association and underlying mechanisms.

### 4.6 Differential metabolites

Circulating cholesterol is the major metabolic risk of NAFLD patients, with the potential to develop cardiovascular diseases (131, 132). Both low-density lipoprotein (LDL) and high-density lipoprotein (HDL) are carriers of cholesterol, with LDL moving cholesterol into the arteries and HDL clearing cholesterol from the arteries. Dyslipidemia is a common complication of NAFLD (133). Cheng et al. (9) found that among 394 NAFLD patients (16. 5% lean), lean NAFLD patients are older, more female and have higher levels of HDL, but lower serum triglyceride and alanine transaminase (ALT) levels in contrast to non-lean patients. Compared with the lean healthy group, lean NAFLD patients showed a higher percentage of hyperlipidemia, age, and an increase in waist circumference, serum triglyceride, LDL-C, and blood glucose levels (9). A study involving 1,305 Chinese residents found that sarcopenic lean NAFLD patients presented a distinct metabolomic profile that is prone to carotid plaque and liver fibrosis, with increased serum valine, small LDL triglyceride and VLDL5 components, and reduced components of HDL4 (134). Park et al. found that higher relative grip strength, one of the indicators of sarcopenia, is associated with lower TG, TC, and LDL-C and higher HDL-C levels (135). These studies suggest that LDL-C, TG and TC as well as HDL-C might be biomarkers of sarcopenic lean NAFLD.

Bile acids (BAs) are amphiphilic steroid molecules synthesized from cholesterol in the liver (136). BAs regulate lipid and glucose metabolism, as well as the synthesis, transport and reabsorption of BAs through bile acid receptors (137). The dysfunctional BA metabolism promotes the accumulation of fat and infiltration of inflammatory cells in the liver (4). Compared with non-lean NAFLD patients, the total BA, primary BA and secondary BA levels are higher in lean NAFLD patients. BA profiling showed that lean NAFLD patients had lower levels of deoxycholic acid (DCA), glycodeoxycholic acid (GCDCA) and goosenodeoxycholic acid (CDCA), and higher levels of glycocholic acid (GCA) (3). Studies reported that serum cholic acid (CA), ursodeoxycholic acid (UDCA) and DCA impair muscle fiber structure and function and induce sarcopenia (138–140). And the level of CA is significantly higher in patients with severe fibrosis compared with those with none/mild fibrosis (3).

Serum uric acid, a metabolite of purines in the liver, is considered to be a predictor of insulin resistance and the severity of liver injury in NAFLD (141). Serum uric acid levels are reported to be higher in lean NAFLD than in healthy controls, but HDL-c levels are in the contrary. Since the ratio of uric acid to HDL-c (UHR) is independently associated with an increased risk of NAFLD, UHR might be a new promising marker for lean NAFLD (142). Some studies have reported a positive association between serum uric acid levels and relative grip strength (143–145). However, a cross-sectional study of 5,247 adults from Korea found a negative association (146). Therefore, the relationship between serum uric acid level and sarcopenia remains controversial and needs further studies to verify the association.

### 4.7 Altered gut microbiota

The gut microbiome has a significant contribution to digestion, vitamin synthesis and pathogen resistance (147). The disruption of intestinal homeostasis and changes in microbiota are involved in the pathogenesis of NAFLD (147). Recent studies highlight that lean NAFLD subjects have a distinct gut microbiota profile from obese ones (18).

In a study conducted in a Chinese population, lean NAFLD subjects demonstrated a reduced enrichment of *Firmicutes*, including *Lachnospiraceae, Ruminococcaceae, Lactobacillaceae*, and an increase in lipopolysaccharide-producing *Gram-negative* bacteria (148). Chen et al. (3) reported a distinct microbiome profile in a Caucasian population, and found that lean NAFLD patients have an increased proportion of *Ruminococcaceae* compared to obese NAFLD patients and an increased population of *Dorea* and decreased populations of *Marvinbryantia* and *Christensellenaceae* compared with healthy controls. According to the analysis of liver biopsy proved NASH patients, intestinal permeability and bacterial overgrowth are associated with the severity of steatosis (149). The gut microbiomes affect FXR-mediated signaling pathways in the liver and gut by interacting with BAs, which is associated with the pathogenesis of lean NAFLD (150, 151). Gut microbiome and BA alterations may predispose NAFLD development at a lower BMI, and further studies of microbiome modulation are required to better understand its role as both a potential biomarker and a therapeutic option in lean NAFLD patients (152).

Nikkhah et al. found that the alteration in age-related sarcopenia and liver cirrhosis-induced sarcopenia was a reduction in short-chain fatty acids (SCFAs) -producing bacteria. *Lachnospiraceae* family, consisting of *Lachnospira, Fusicatenibacter, Roseburia*, and *Lachnoclostridium*, is significantly decreased in age-related sarcopenia, while in liver cirrhosis-induced sarcopenia, the alpha diversity of gut microbiota decreased in comparison to the control group (153). The richness of gut microbiota is considerably reduced in sarcopenic patients, and the *Firmicutes/Bacteroidetes* ratio, *Agathobacter, Dorea* and *Butyrate* are decreased, whereas *Shigella* and *Bacteroides* are enriched in the gut (154). A case-control study also reported that *Phylum Bacteroides* is significantly decreased in old-women with sarcopenia, whereas genus *Prevotella* is increased (155). However, the relationship between the types and richness of gut microbiota in sarcopenia and lean NAFLD is not clear.

## 5 Strategies for sarcopenia and lean NAFLD

There is a close relationship between the amount of physical activity and mortality, and people with more physical activity have a lower risk of death (156). Lack of physical activity leads to loss of muscle mass and reduced energy expenditure, leading to obesity and hepatic steatosis (157). Chronic inflammation, oxidative stress, and insulin resistance can worsen sarcopenia and NAFLD (158). During exercise, the production of pro-inflammatory cytokines decreased while the anti-inflammatory cytokines and muscle protein synthesis, as well as glucose uptake increased. Physical activity may reduce the risk of sarcopenia progression (159). Studies found that exercise improves metabolic health even without observable weight loss (160, 161).

Circuit training has been reported as an effective way to simultaneously improve both the muscular and cardiovascular systems. Circuit training programs offer a combination of aerobic and resistance exercises, the intensity of which can be adapted to the individual and purpose. In a Korean study on circuit training intervention on elderly women with sarcopenia, the muscle mass and strength, body composition, balance, and pulmonary function in subjects were improved after 12-week circuit exercise training (162). Nutrition supplements also have a significant contribution to the improvement of sarcopenia. A randomized controlled trial shows that the skeletal muscle mass index in the exercise and protein supplementation group is significantly higher than either the exercise-only or protein supplementation-only groups. Simultaneously, the increase in grip strength and gait speed is significantly greater for the exercise and protein supplementation group than for the protein supplementation-only group (163). Other trials also proved that nutrition such as amino acids, vitamin D, or calcium supplements improve muscle mass, strength, and physical function in patients with sarcopenia (164–168). The above evidence indicates that combined intervention of exercise (including aerobic and resistance exercises) and nutrition (such as whey protein, branched-chain amino acids, and vitamin D as well as calcium supplement) has positive effects on sarcopenia, which provide a theoretical basis and therapeutic strategy for the improvement of sarcopenia. Since sarcopenia is associated with some hormones disorder and decreased muscle protein synthesis, some drugs such as sex hormones (e. g. testosterone and 17 beta-estradiol plus cyclic norethisterone acetate) and monoclonal antibodies that stimulate muscle growth (e. g. bimagrumab) have been used to improve sarcopenia (169–171).

Currently, there is no specific drug for the treatment of NAFLD including lean NAFLD. However, some potential medications might be suitable for countering the syndromes or preventing disease progression. Insulin resistance is one of the most critical potential co-pathogenesis of sarcopenia and lean NAFLD (48). Pioglitazone is a PPAR-γ agonist that increases insulin sensitivity, and exerts anti-inflammatory and anti-atherosclerosis effects (172). In the UTHSCSA NASH Phase 4 trial (NCT00994682) (173), the number of patients in the pioglitazone group who recovered from impaired fasting glucose (IFG)/impaired glucose tolerance (IGT) is significantly higher than that in the placebo group, suggesting that pioglitazone has a desirable effect in reducing metabolic risk factors, which can be used as a potential therapeutic agent for lean NAFLD. Dapagliflozin and Empagliflozin, sodium-glucose cotransporter 2 (SGLT-2) inhibitors, are approved by the FDA as oral hypoglycemic agents in patients with T2DM (174). A Phase 2 trial (175) studied the effect of Dapagliflozin on NAFLD patients with T2DM, and showed a significant radiographic improvement in liver fat content compared with placebo. SGLT-2 inhibitors have also found to reduce visceral fat, improve glycemic disorder, and protect the heart, which may have therapeutic potential for lean NAFLD (175). Fenofibrate is a drug for dyslipidemia (176). Abdelmoneim et al. (177) fed experimental mice palm oil and fructose to establish a non-obese NAFLD model, and found that fenofibrate could prevent the occurrence of NAFLD, improve high blood glucose and oxidative stress status, indicating that fenofibrate can be a potential candidate for lean NAFLD.

## 6 Conclusions

**Figure 1:**
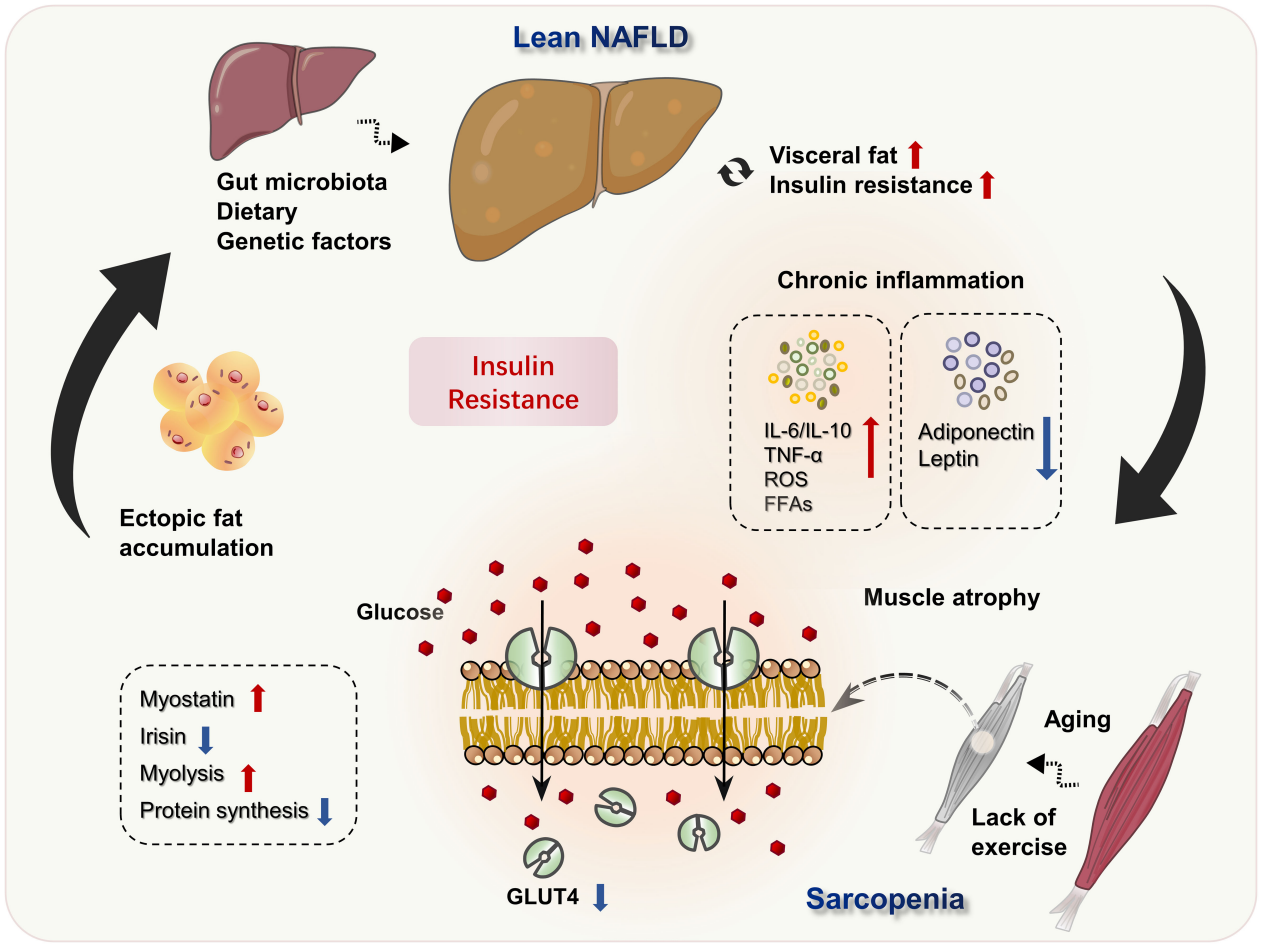
Potential mechanisms of lean NAFLD and sarcopenia. Excessive dietary intake and decreased energy consumption, as well as genetic predisposition and other factors, result in visceral fat accumulation, IR and the development of lean NAFLD. Simultaneously, IR and metabolic inflammatory worsen the living environment of muscle cells, aggravates muscle atrophy, and leads to the occurrence of sarcopenia. Loss of skeletal muscle mass inhibits the GH/IGF-1 axis, resulting in decreased muscle protein synthesis, increased lipolysis. The decreased expression of GLUT4 in sarcopenia, further suppressed glucose utilization and aggravated IR, forming a vicious circle.

Although BMI is within the normal range, lean NAFLD is not a benign state. It can also progress to NASH and fibrosis, and even cirrhosis and hepatocellular carcinoma as obese NAFLD. Lean NAFLD patients even have a higher risk of metabolic syndrome and all-cause mortality in comparison to obese NAFLD patients. Skeletal muscle is a major metabolic organ, and the decline of skeletal muscle quality and quantity leads to a decrease of insulin sensitivity and metabolic disorders. Sarcopenia is commonly coexistent with lean NAFLD, may present in the early stage of liver disease and worsen with the severity of liver disease. Under the background of co-pathogenesis of insulin resistance and chronic inflammation, the interaction between sarcopenia and lean NAFLD forms a vicious circle (**Figure 1**). Clinical interventions should be considered for patients with lean NAFLD as intensively as for those with obese NAFLD, especially when they have a comorbidity of sarcopenia. The first-line recommendation for lean NAFLD patients with sarcopenia is exercise, nutrition supplements and reduce total calories, carbohydrates, and fat as well as total cholesterol, so as to reduce visceral fat and increase skeletal muscle mass, so as to restore the homeostasis of lipid, glucose, endocrinal and metabolic status. However, there is no study to compare the status of sarcopenia between lean NAFLD and obese NAFLD, or exploring the underlying mechanisms, and more studies are needed to explore the pathophysiology and underly mechanisms of lean NAFLD and sarcopenia, which will assist in treatment and preventive strategies for lean NAFLD.

## Author contributions

LZ conceptualized the manuscript, MC collected the literature and drafted the manuscript, YC revised the manuscript and prepared the figure, LZ and GJ revised the manuscript. All authors edited, revised, and approved the final version of this review.
